# Whole exome sequencing identifies ATRX mutation as a key molecular determinant in lower-grade glioma

**DOI:** 10.18632/oncotarget.689

**Published:** 2012-10-11

**Authors:** Kasthuri Kannan, Akiko Inagaki, Joachim Silber, Daniel Gorovets, Jianan Zhang, Edward R. Kastenhuber, Adriana Heguy, John H. Petrini, Timothy A. Chan, Jason T. Huse

**Affiliations:** ^1^ Department of Pathology, Memorial Sloan-Kettering Cancer Center, New York, NY, USA; ^2^ Department of Molecular Biology, Memorial Sloan-Kettering Cancer Center, New York, NY, USA; ^3^ Department of Radiation Oncology, Memorial Sloan-Kettering Cancer Center, New York, NY, USA; ^4^ Department of Human Oncology and Pathogenesis Program, Memorial Sloan-Kettering Cancer Center, New York, NY, USA

**Keywords:** glioma, astrocytoma, IDH, ATRX, whole-exome sequencing

## Abstract

The molecular foundations of lower-grade gliomas (LGGs)—astrocytoma, oligodendroglioma, and oligoastrocytoma—remain less well characterized than those of their fully malignant counterpart, glioblastoma. Mutations in isocitrate dehydrogenase 1 and 2 (IDH1/2) likely represent initiating pathogenic events. However, while IDH mutations appear to dramatically alter cellular epigenomic landscapes, definitive downstream transformative mechanisms have not been characterized. It remains likely, therefore, that additional genomic abnormalities collaborate with IDH mutation to drive oncogenesis in LGG. We performed whole exome sequencing in 4 LGGs, followed by focused resequencing in an additional 28, and found a high incidence of mutations in the ATRX gene (α thalassemia/mental retardation syndrome X-linked). ATRX forms a core component of a chromatin remodeling complex active in telomere biology. Mutations in ATRX have been identified in multiple tumor types and appear to cause alternative lengthening of telomeres (ALT), a presumed precursor to genomic instability. In our samples, ATRX mutation was entirely restricted to IDH-mutant tumors, closely correlated with TP53 mutation and astrocytic differentiation, and mutually exclusive with 1p/19q codeletion, the molecular hallmark of oligodendroglioma. Moreover, ATRX mutation was highly enriched in tumors of so-called early progenitor-like transcriptional subclass (~85%), which our prior work has linked to specific cells of origin in the forebrain subventricular zone. Finally, ATRX mutation correlated with ALT, providing a mechanistic link to genomic instability. In summary, our findings both identify ATRX mutation as a defining molecular determinant for a large subset of IDH-mutant gliomas and have direct implications on pathogenic mechanisms across the wide spectrum of LGGs.

## INTRODUCTION

Diffuse gliomas represent a biologically heterogeneous group of primary brain tumors whose shared propensity to widely infiltrate surrounding brain parenchyma renders them incurable, even in the face of ionizing radiation and cytotoxic chemotherapy [1]. Recent comprehensive genomic profiling has greatly clarified the molecular foundations of glioblastoma (GBM, WHO grade IV) the most common and malignant diffuse glioma variant [2-6], and promoted the exploration of an array of therapeutic strategies [7, 8]. By contrast, the underlying pathogenic events driving lower-grade diffuse gliomas (LGGs, WHO grade II and III) are considerably less clear.

Current convention dictates that LGGs be designated as either astrocytoma, oligodendroglioma, or mixed glioma (oligoastrocytoma), primarily on the basis of histopathological criteria [9]. Recently, point mutations in the isocitrate dehydrogenase genes *IDH1* and *IDH2* have been identified in 70-90% of LGGs spanning across all morphologic subtypes [10-12]. IDH mutations induce a neomorphic enzymatic activity that preferentially generates the oncometabolite R(–)-2-hydroxyglutarate at the expense of the normal TCA cycle component α-ketoglutarate [13, 14]. And while this process appears to cause widespread disruptions in cellular physiology, particularly with respect to the epigenome [15, 16], the precise mechanisms by which IDH mutation promotes tumorigenesis remain to be elucidated. In this setting, it is highly likely that additional molecular aberrations conspire with IDH mutation to induce tumorigenesis.

It has been known for some time that coordinated loss of chromosomes 1p and 19q (1p/19q codeletion) designates a prognostically favorable LGG subgroup comprised primarily of oligodendrogliomas and, to a lesser extent, oligoastrocytomas, while largely excluding astrocytomas [17-19]. More recent work has demonstrated an almost invariable association between 1p/19q codeletion and IDH mutation, implying that both abnormalities are required for transformation in tumors of fundamentally oligodendroglial lineage [11]. By contrast, the molecular landscape of LGGs harboring intact 1p/19q—predominantly astrocytomas—appears less uniform, with mutations in *TP53* representing their single most prevalent genomic anomaly apart from IDH mutation [11, 20]. To gain a better understanding of this heterogeneity, we recently employed global transcriptional analysis to establish three molecularly and clinically distinct subclasses within astrocytic (1p/19q-intact) LGGs [21]. One group, termed preglioblastoma (PG), consisted primarily of IDH-wild type tumors while the other two, termed neuroblastic (NB) and early progenitor-like (EPL), were almost exclusively IDH-mutant. These latter two subclasses were further delineated by different rates of *TP53* mutation and their associations, by way of gene expression patterns, to distinct neuroglial precursor cell pools. Definitively linking these transcriptional subgroupings with specific pathogenic mutations would be of considerable interest.

To further explore the genomic landscape of LGG, particularly with reference to its different histopathological variants, we performed whole exome capture, next-generation sequencing on 4 WHO grade II gliomas followed by validation sequencing in an additional 28. We found a strikingly high incidence of mutations in the *ATRX* gene (α thalassemia/mental retardation syndrome X-linked), entirely restricted to IDH-mutant LGGs of astrocytic lineage—astrocytomas and oligoastrocytomas—and mutually exclusive with 1p/19q codeletion. ATRX and its binding partner DAXX (death-associated protein 6) are central components of a chromatin remodeling complex required for the incorporation of H3.3 histone proteins into the telomeric regions of chromosomes [22, 23]. Dysfunction of the ATRX/DAXX complex results in a phenomenon known as alternative lengthening of telomeres (ALT) along with more widespread genomic destabilization [24-26]. Mutations in *ATRX* and, to a lesser extent, *DAXX* have recently been identified in a number of tumor subtypes, including pediatric and adult gliomas [24, 26-29]. In this report, we show that *ATRX* mutation is present in ~70% of IDH-mutant, 1p/19q intact LGGs. Moreover, we demonstrate that *ATRX* mutation represents the defining molecular abnormality delineating the EPL subclass of LGG from other IDH-mutant astrocytic tumors. Finally, we correlate *ATRX* mutation with ALT, recapitulating a functional association with mitotic instability seen in other *ATRX*-mutant tumor types.

## RESULTS

### High-throughput resequencing demonstrates a high rate of ATRX mutation in IDH-mutant, 1p/19q-intact LGGs

To identify molecular abnormalities conspiring with IDH mutation in the pathogenesis of LGGs, we performed whole exome capture, next-generation sequencing on genomic DNA extracted from 4 WHO grade II gliomas—3 astrocytomas and 1 oligoastrocytoma—with known mutations in *IDH1*. Additionally, all 4 tumors were 1p/19q-intact. DNA from case-matched blood was also sequenced to ascertain and screen out germline polymorphisms. We achieved a mean coverage of 57-fold in targeted regions, with 80% of bases represented by at least 10 reads (Table S1). After quality filtering (see Material and Methods) our analysis revealed 376 candidate somatic mutations in 339 genes, which consisted of missense (85%), nonsense (4%), splice site (5%), and insertion/deletion (4%) variants (Table S2). We found that C:G to T:A transitions predominated, as has been reported for several other cancer subtypes [30] (Tables S3-S4).

We assembled promising candidate mutations based on coverage, mutational frequency, read quality, and predicted functional impact (see Materials and Methods for precise criteria) and subjected them to bi-directional Sanger sequencing validation. The resulting list of confirmed mutations demonstrated only 3 genes altered in more than one tumor: *IDH1, TP53*, and *ATRX* (Table 1). *ATRX* mutation was present in 3 of 4 tumors, representing the most frequent genomic alteration in our sample set apart from IDH mutation.

**Table 1:** Validated somatic mutations from whole exome sequencing of 4 IDH-mutant LGGs

Gene	Transcript Accession	Tumor	Amino Acid Change
IDH1	NM_005896.2	G4	p.R132H
		G5	p.R132G
		G18	p.R132H
		G6	p.R132H
ATRX	NM_000489.3	G5	p.K1001fs
		G18	p.R1426X
		G6	p.L639fs
TP53	NM_000546.5	G5	p.R273C
		G6	p.R273C
MUC4	NM_018406.6	G5	p.D1413N
MYBBP1A	NM_001105538.1	G4	p.K277N
OR2B6	NM_012367.1	G18	p.M118V
ABCC11	NM_032583.3	G18	p.V668M
GABRB3	NM_000814.5	G5	p.P145L
TNFRSF11B	NM_002546.3	G18	p.L331M
CR1	NM_000651.4	G6	p.V1927L
KIAA1731	NM_033395.1	G5	p.L272P
HERC2	NM_004667.5	G5	p.S2763C
KIAA1211	NM_020722.1	G5	p.A1105V
EXT2	NM_000401.3	G5	p.N131K
SRA1	NM_001035235.3	G5	p.V110delinsGL

To better determine the incidence of *ATRX* mutation across LGG subtypes, we performed high-throughput resequencing of all *ATRX* exons in 28 additional LGGs, along with case-matched blood. Our combined sample set consisted of 13 astrocytomas (8 WHO grade II and 5 WHO grade III), 12 oligodendrogliomas (6 WHO grade II and 6 WHO grade III), and 7 oligoastrocytomas (all WHO grade II). The coding regions of *DAXX*, *TP53*, *IDH1*, and *IDH2* were also subjected to resequencing and results were correlated with known 1p/19q status from de-identified patient records (Table 2). Once again, all candidate mutations were validated by Sanger sequencing. We found that *ATRX* mutations were present in 12 tumors, all of which also harbored *IDH1* mutations. By contrast, *ATRX* was uniformly wild type in the 4 IDH-wild type gliomas featured in our tumor cohort. *DAXX* mutations were not identified in either group. The pattern of *ATRX* mutations exhibited remarkable associations with other genomic abnormalities within IDH-mutant LGGs. For instance, *ATRX* mutation was significantly correlated with *TP53* mutation (*P*=0.0189) and anticorrelated with 1p/19q codeletion to the point of complete mutual exclusivity (*P*=0.0003). All told, *ATRX*-mutant tumors accounted for ~70% of IDH-mutant, 1p/19q-intact LGGs in our sample set. Given these findings, we were not surprised that *ATRX* mutation was also strongly linked to astrocytic and oligoastrocytic morphology, as opposed to pure oligodendroglial morphology (*P*=0.0009). No significant correlations were found between *ATRX* mutation and either patient age, primary/recurrent tumor status, or tumor grade. Similar to that seen previous reports [24, 26, 27], the distribution of *ATRX* mutations identified in our sample set was broad, spread across more than half of the protein, and consisted of frameshift (6), nonsense (2), missense (1), and in-frame deletion (3) variants. There were no readily apparent associations between the type or location of specific *ATRX* mutations and other genomic, demographic, or histopathological parameters, although our sample size likely precluded robust analysis in this regard.

**Table 2:** **Validated sequencing results for 32 LGGs showing mutations in *IDH1, ATRX, and TP53***.1p/19q status, astrocytoma transcriptional subclass [21], and ALT FISH results are also shown along with patient age, primary/recurrent tumor status, and pathological diagnosis. WT: wild type, A: astrocytoma, O: oligodendroglioma, OA: oligoastrocytoma, II: WHO grade II, III: WHO grade III, NB: neuroblastic, EPL: early progenitor-like, PG: preglioblastoma

Tumor	Age	Primary/Recurrent	IDH1	ATRX	TP53	Pathology	1p/19q	Astro Subclass	ALT FISH
G1	56	Primary	p. R132H	WT	p.R141C	A II	intact	NB	NEG
G2	27	Recurrent	p. R132H	WT	WT	OA II	intact	NB	NEG
G3	41	Primary	p. R132H	WT	p.P90fs	OA II	intact	NB	POS
G4	31	Primary	p. R132H	WT	WT	A II	intact	NB	NEG
G5	34	Primary	p. R132G	p.K1001fs	p.R141C	A II	intact	EPL	POS
G6	40	Primary	p. R132H	p.L639fs	p.R141C	A II	intact	EPL	POS
G7	35	Primary	p. R132H	p.E991fs	p.G196A	A II	intact	EPL	POS
G8	38	Primary	p. R132H	p.R907X	p.R141C	OA II	intact	EPL	POS
G9	41	Primary	p. R132H	p.1338_1339del	WT	A II	intact	EPL	NEG
G10	29	Primary	p. R132C	p.1346_1347del	WT	OA II	intact	EPL	POS
G11	58	Recurrent	p. R132H	p.E1010fs	WT	OA II	intact	EPL	POS
G12	38	Recurrent	p. R132H	WT	p.L232R	A III	intact	EPL	NEG
G13	43	Recurrent	p. R132H	p.199_205del	p.R141C	A II	intact	EPL	POS
G14	52	Primary	p. R132H	p.K1018fs	p.R141C	A III	intact	EPL	POS
G15	36	Primary	p. R132H	p.R1302fs	p.101_103del	A III	intact	EPL	POS
G16	34	Primary	p. R132H	p.R221K	p.G196X	A III	intact	EPL	POS
G17	36	Primary	WT	WT	WT	A III	intact	EPL	NEG
G18	32	Primary	p. R132H	p.R1426X	WT	OA II	intact	PG	POS
G19	62	Primary	WT	WT	WT	A II	intact	PG	NEG
G20	65	Primary	WT	WT	WT	O III	intact	PG	NEG
G21	34	Recurrent	p.R132H	WT	WT	OA II	codeleted	N/A	NEG
G22	41	Recurrent	p.R132H	WT	WT	O II	codeleted	N/A	N/A
G23	38	Primary	p.R132H	WT	WT	O II	codeleted	N/A	NEG
G24	37	Primary	p.R132H	WT	WT	O III	codeleted	N/A	NEG
G25	52	Recurrent	p.R132H	WT	WT	O II	codeleted	N/A	NEG
G26	34	Recurrent	p.R132H	WT	WT	O II	codeleted	N/A	NEG
G27	45	Primary	p.R132H	WT	WT	O II	codeleted	N/A	NEG
G28	37	Primary	p.R132H	WT	WT	O II	codeleted	N/A	NEG
G29	62	Primary	p.R132H	WT	WT	O III	codeleted	N/A	NEG
G30	46	Primary	p.R132H	WT	WT	O III	codeleted	N/A	NEG
G31	35	Recurrent	p.R132H	WT	WT	O III	codeleted	N/A	NEG
G32	58	Primary	WT	WT	WT	O III	codeleted	N/A	NEG

### ATRX mutation defines the early progenitor-like molecular subclass of astrocytoma and correlates with ALT

**Figure 1:**
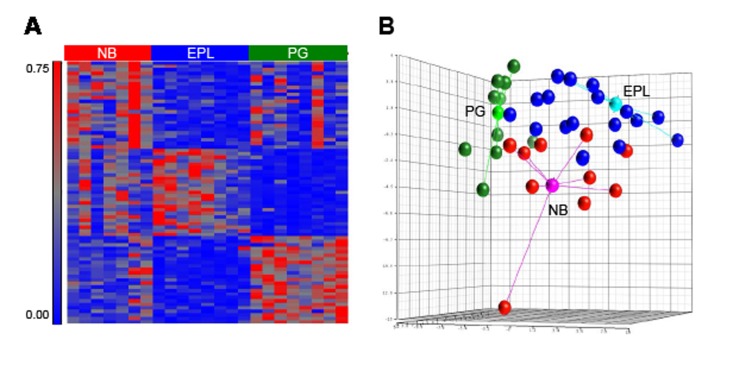
Nanostring classifier designates transcriptional subclasses of astrocytic LGG A, standardized intensity plot showing 75-gene classifying signature applied to 23 training samples of known subclass. B, Nanostring profiling for training set and classified samples visualized by three dimensional principal component analysis. Centroids for training set samples are shown along with connectors. NB: neuroblastic, EPL: early progenitor-like, PG: preglioblastoma

As indicated above, our earlier work designated 3 molecularly and clinically distinct subclasses of astrocytic LGGs. To determine the composition of *ATRX* mutations within each subclass, we developed a multiplexed mRNA profiling assay on the Nanostring platform consisting of a 75-gene signature (25 upregulated genes per subclass) and applied it to total RNA derived from 1p/19q-intact tumors in our sample set. Raw data was then processed through a classifying algorithm based on Linear Discriminant Analysis and trained on material from 24 astrocytic tumors of known subclass—7 NB, 8 EPL, and 8 PG (FIG. 1A-1B). Additionally, earlier work had already established the subclass assignment of 4 gliomas in our sequencing cohort [21].

We found a striking association between *ATRX* mutation and EPL subclass (*P*=0.0044) (Table 2). In total, 85% (11/13) of astrocytic LGGs designated as EPL by expression profiling harbored *ATRX* mutations, and 92% (11/12) of *ATRX* mutations were found in EPL tumors. Taken together, these findings indicate that *ATRX* mutation itself represents the defining molecular abnormality of a distinct astrocytoma subclass delineated by gene expression.

**Figure 2:**
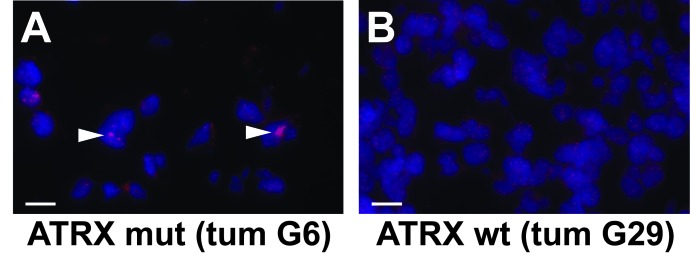
Telomere FISH demonstrates ALT in *ATRX*-mutant LGG Representative micrographs showing telomere FISH (red) performed in *ATRX*-mutant (mut; A) and wild type (wt; B) LGGs. An intranuclear ALT-positive focus is indicated (arrowhead). Slides were counterstained with DAPI (blue). White scale bars are 120 μm.

Previous studies have directly linked *ATRX* mutation to ALT and genomic instability in a variety of tumors [24-26]. To assess whether ALT correlates with *ATRX* mutational status in LGG, we performed telomere FISH on our sample set. Consistent with earlier reports, we found a notably strong association between *ATRX* mutation and ALT (*P*<0.0001), as indicated by ultrabright foci of intranuclear positivity by telomere FISH in multiple tumor cells (FIG. 2A-2B; Table 2). In total, 11/12 (92% of LGGs with *ATRX* mutations exhibited ALT, and 11/12 (92%) of LGGs with ALT harbored *ATRX* mutations.

## DISCUSSION

While the natural history of LGGs almost invariably culminates in their transformation to WHO grade IV tumors, their tendency to exhibit prolonged periods of indolent growth sharply contrasts with the biological behavior of GBMs [1]. This relative clinical stability likely reflects a less fundamentally altered physiological state, and provides an inviting “window of opportunity” for the implementation of appropriately targeted therapeutics. Elucidating the precise mechanisms driving LGG pathogenesis, therefore, is of vital importance to the advancement of their clinical management. The sheer frequency of IDH mutation in LGG demonstrates its biological relevance and highlights the unequivocal importance of epigenomics and metabolomics in gliomagenesis. Yet while its physiological effects are broad and profound, exactly how IDH mutation fundamentally drives a neoplastic phenotype remains unclear. Indeed, the inability of IDH mutation to promote glioma formation in mouse models thus far underscores this quandary, and further implies that additional molecular alterations are likely required for transformation.

We employed both whole exome and targeted next-generation sequencing approaches to identify *ATRX* mutations in a significant percentage of LGGs. Intriguingly, the distribution of *ATRX* mutations tracked with specific diagnostic and molecularly defined tumor subclasses. Specifically, their presence was entirely restricted to IDH-mutant, 1p/19q-intact LGGs, astrocytic and oligoastrocytic in their morphology, where they were found in ~70% of tumors and exhibited a tight correlation with *TP53* mutation. These findings are strikingly consistent with those of a very recent study examining, among other parameters, *ATRX*, *TP53*, and *IDH* mutational status, along with 1p/19q codeletion in a large cohort of low- and high-grade gliomas [28]. By contrast, a separate report, also very recent, found a somewhat lower rate of *ATRX* abnormalities in astrocytic and oligoastrocytic LGGs (~42%) [29]. This discrepancy likely reflects the latter study's employment of immunohistochemical staining in tissue microarrays, and not sequencing-based mutational analysis, as its primary screening technique for ATRX abnormalities. Indeed, frequent ATRX mutations (~79%) were revealed in a smaller cohort of tumors actually subjected to sequencing in this same study. Regardless, together with ours, both reports support a fundamental molecular stratification of LGGs based on *IDH* and *ATRX* mutational status and 1p/19q codeletion.

Additionally, our data indicate that abnormalities in *ATRX* represent the defining molecular characteristic of the EPL subclass of astrocytic LGGs. We considered the possibility that functional loss of ATRX, a known chromatin regulator, might itself drive the EPL transcriptional signature. However, the recent finding that ATRX loss does not significantly alter H3.3 profiles across the coding genome argues against this [22], and suggests a more fundamental association between EPL subclass and specific cells of origin for IDH-mutant LGG. Our previous work established biological links between EPL tumors and early-stage neuroglial precursor cells in the mammalian subventricular zone (SVZ) [21]. The high frequency of *ATRX* mutations in EPL tumors, therefore, implies either a unique propensity of this specific SVZ progenitor population to acquire *ATRX* mutation or a particular sensitivity to its biological effects. Indeed, it has recently been suggested that regions harboring stem-like cells in the adult brain, including the SVZ, might be inherently more prone to oncogenic initiating events [31].

The invariable co-occurrence of *ATRX* mutations with IDH mutations, and their frequent association with *TP53* mutation support a cooperative pathogenic mechanism by which dysfunction in all three proteins is required for oncogenesis in a large subset of LGG. Recent work has shown that IDH mutation dramatically reprograms the cellular epigenome, the physiological effects of which include impaired differentiation and the abnormal maintenance of stem and progenitor cell populations in physiological states permissive to self-renewal [15, 16, 32]. Additionally, multiple studies have demonstrated that loss of ATRX protein or *ATRX* mutation results in ALT and genomic instability [24-26, 28], and our own findings recapitulate these functional relationships in IDH-mutant LGGs. Combining genomic instability with inherent self-renewal potential could provide fertile ground for malignant transformation, particularly in the setting of *TP53* loss, which would presumably allow affected cells to evade apoptosis and/or senescence [33]. More extensive *in vitro* and *in vivo* modeling in disease-relevant experimental systems will be essential to test the validity of these and other related conjectures.

The recent identification of H3.3 histone protein mutations in pediatric GBM and diffuse intrinsic pontine glioma (DIPG) is particularly intriguing in light of our present findings [26, 34, 35]. Specifically, their frequent association with *ATRX* and *TP53* mutations suggests a functional equivalence with IDH mutation in adult glioma, with both fundamentally altering global epigenomic landscape and cellular differentiation state. Similarly, the mutual exclusivity of *ATRX* mutation with 1p/19q deletion in adult IDH-mutant LGGs also implies analogous functionality, perhaps in the mediation of genomic instability. Thus, accruing evidence from direct molecular profiling in tumor tissue has bolstered the notion of a shared pathogenic mechanism, operative across a wide spectrum of glioma subtypes. While much work remains to be done, the detailed functional characterization of a common transformative pathway in LGG would have significant implications on future therapeutic development and disease management.

## METHODS

### Glioma sample cohort

Fresh frozen glioma tissue and de-identified demographic and clinical data was obtained from Memorial Sloan Kettering Cancer Center (MSKCC) Brain Tumor Center (BTC) following approval from the Institutional Review Board under the auspices of a blanket biospecimen utilization protocol. Frozen samples were mounted, sectioned, and stained with hematoxylin and eosin (H& E) before being examined by a Neuropathologist (J.T.H.) for tumor adequacy. Specimens passing quality control were processed for genomic DNA and total RNA extraction using commercially available reagents (Qiagen, Valencia, CA, USA).

### High-throughput sequencing

Whole exome capture of 4 LGG samples consisting of matched tumor/normal pairs was performed using the SureSelect 50MB Target Enrichment System (Agilent Technologies, Santa Clara, CA, USA) according to the manufacturer's standard protocol. Libraries were then subjected to 50 bp + 35 bp paired-end sequencing on the SOLiD 5500xl platform (Applied Biosystems, Foster City, CA, USA). Sequencing reads were aligned to the reference human genome (hg19) using the BWA aligner (open source software). Somatic mutations were then identified using an automated computational pipeline (Mut(e)Piper). In its first stage, the pipeline consists of GATK [36] base quality score recalibration, indel realignment, and duplicate removal using standard parameters [37]. Mut(e)Piper then employs SomaticSniper [38] and Somatic Indel Detector (GATK) to identify single nucleotide variants (SNVs) and insertions/deletions. This process identified a total of 19883 somatic variants in our initial 4 sample pairs, ranging from 4329 to 6601 per tumor with an average of 4971 (Table S5). The data were filtered using cutoffs of 3% allelic frequency for the variant allele in tumor and 97% allelic frequency for the reference (hg19) allele in normal. The candidate variant list was then further pruned using 1000genomes, dbSNP132 and ESP5400 (NHLBI Exome Sequencing Project (ESP), Exome Variant Server) databases to exclude confirmed single nucleotide polymorphisms. Short-listed variants were annotated using ANNOVAR (UCSC Known Genes) [39] and somatic mutations were identified as non-synonymous substitutions and frameshift/non-frameshift deletions/insertions. To assemble a list of high-value mutations for initial validation, we selected all recurrent mutations and non-recurrent mutations meeting the following criteria: 1) 20X and 9X coverage in normal and tumor samples respectively, 2) an average regional (100 bp) phred mapping quality score of >15, 3) presence of mutations on nonduplicated reads, 4) mutational allelic frequency of >15, and 4) a MutationAssessor [40] functional impact score of >1. These filters produced a candidate list of 43 mutations involving 27 genes (Table S6) that were then subjected to Sanger Sequencing validation (see below). An expanded cohort of 28 additional tumors/normal pairs was subjected to focused resequencing for *ATRX*, *IDH1/2*, *TP53*, and *DAXX* on the MiSeq platform (Illumina, San Diego, CA, USA). Exonic and intron-exon border regions for each gene were captured with a custom NimbleGen SeqCap EZ bait library for short reads (Roche, Madison, WI, USA), following the manufacturer's standard protocol. Mean coverage for captured regions ranged from 10 to 134 reads with an average of 86 (Table S7). Sequencing data was processed using the Mut(e)Piper pipeline (see above).

### Sanger sequencing

Exons of interest (NCBI Human Genome Build 36.1) were broken into amplicons of 350 bp or less, and specific primers were designed using Primer3 (http://frodo.wi.mit.edu/primer3/) to cover exonic regions and ~50 bp of flanking intronic sequence. PCR reactions were carried out in 384 well plates, in a Duncan DT-24 water bath thermal cycler, with 10 ng of template DNA (Repli-G Midi, Qiagen) using a touchdown PCR protocol with Kapa2G Fast HotStart Taq (Kapa Biosystems, Cape Town, South Africa) consisting of 1 cycle of 95°C for 5 min; 3 cycles of 95°C for 30 sec, 64°C for 15 sec, 72°C for 30 sec; 3 cycles of 95°C for 30 sec, 62°C for 15 sec, 72°C for 30 sec; 3 cycles of 95°C for 30 sec, 60°C for 15 sec, 72°C for 30 sec; 37 cycles of 95°C for 30 sec, 58°C for 15 sec, 72°C for 30 sec; 1 cycle of 70°C for 5 min. Templates were purified using AMPure (Agencourt Biosciences, Beverly, MA, USA), split into two, and sequenced bi-directionally with M13 forward and reverse primers using the Big Dye Terminator Kit v.3.1 (Applied Biosystems) at Agencourt Biosciences. Dye terminators were removed using the CleanSEQ kit (Agencourt Biosciences), and sequence reactions were run on an ABI PRISM 3730xl sequencing apparatus (Applied Biosystems).

### Nanostring expression profiling

We developed a Nanostring-based classifier for astrocytic LGGs based on previous work [21]. Briefly, Receiver Operator Characteristic (ROC) rankings for subclass-associated transcripts were used to identify 25 signature genes per subclass (Table S8). A specific CodeSet (Nanostring Technologies, Seattle, WA, USA) was derived for this gene list and applied, using a Nanostring nCounter, to RNA derived from 23 astrocytic LGGs of known subclass. Raw data was subjected to quantile normalization and used to train a Linear Discriminant Analysis classifying algorithm on the Multi-Experiment Viewer (MeV) software platform (www.tm4.org/mev/). Nanostring data from our sequencing cohort was then processed through this pipeline for final tumor subclassification.

### ALT fluorescence in situ hybridization (FISH)

5 μm frozen sections were fixed with 4% paraformaldehyde for 10 minutes, dehydrated, and denatured at 85 °C for 5 minutes in hybridization mixture consisting of 10 mM Tris-HCL pH 7.2, 70% formamide, 0.5% blocking solution reagent (Roche), and a complementary Cy3-labeled peptide nucleic acid probe specific to the mammalian telomere repeat sequence TTAGGGTTAGGGTTAGGG 3' (Applied Biosystems). Hybridization was performed at room temperature for two hours in the hybridization mixture. Slides were then washed twice with 70% formamide, 10 mM Tris-HCl pH 7.0-7.5, 0.1% BSA followed by multiple washes in PBS. Finally, slides were mounted in Prolong Gold with DAPI (Invitrogen, Grand Island, NY, USA) and examined using a Leica CTR5000 inverted fluorescent microscope. ALT was identified by the presence of ultrabright, clumpy, intra-nuclear foci as previously described [24]. Companion H & E stained sections were examined in all cases to localize tumor tissue.

### Statistics

Univariate associations were evaluated using two-tailed Fisher's exact tests. Stated *P* values do not reflect Bonferroni correction. Please see other Materials and Methods subsections for statistical considerations involving specific analytical pipelines.

## Supplementary Tables


