# Supplementary material for: Whole exome sequencing identifies ATRX mutation as a key molecular determinant in lower-grade glioma
Source: Oncotarget. 2012 Oct 11;3(10):1194–203. doi: 10.18632/oncotarget.689 (PMC3717947; doi:10.18632/oncotarget.689)
Supplement: Supplementary file 1 [file oncotarget-03-1194-s001.pdf]

Whole exome sequencing identifies *ATRX* mutation as a key molecular determinant in lower-grade glioma –

Kannan et al

Table S1: **Read number and coverage statistics for whole exome sequencing performed on 4 IDH-mutant LGGs with case-matched normal.**

Metrics	G4 Normal	G4 Tumor	G5 Normal	G5 Tumor	G18 Normal	G18 Tumor	G6 Normal	G6 Tumor	Average
GENOME_SIZE	3095693983	3095693983	3095693983	3095693983	3095693983	3095693983	3095693983	3095693983	
BAIT_TERRITORY	51756122	51756122	51756122	51756122	51756122	51756122	51756122	51756122	
TARGET_TERRITORY	51756122	51756122	51756122	51756122	51756122	51756122	51756122	51756122	
BAIT_DESIGN_EFFICIENCY	1	1	1	1	1	1	1	1	
TOTAL_READS	136140377	124399279	193127054	270696668	211391011	138118163	123083191	120412604	
PF_READS	135154958	123273842	191737610	268247866	209855065	136871604	122201832	119319707	
PF_UNIQUE_READS	112950205	101872365	147766208	176634114	163123238	112582950	103001316	95380885	
PCT_PF_READS	0.992762	0.990953	0.992806	0.990954	0.992734	0.990975	0.992839	0.990924	
PCT_PF_UQ_READS	0.82966	0.818914	0.765124	0.652517	0.771666	0.815121	0.836843	0.792117	
PF_UQ_READS_ALIGNED	95866908	86890718	125056430	150066288	136452209	95909463	87663421	80913663	
PCT_PF_UQ_READS_ALIGNED	0.848754	0.852937	0.846313	0.849588	0.836498	0.8519	0.85109	0.848322	
PF_UQ_BASES_ALIGNED	3916282633	3609846254	5089321241	6214430031	5540181681	3976534633	3573499599	3362697220	
ON_BAIT_BASES	2440842236	2244920309	3201046680	4105730234	3498398004	2511802216	2260953633	2144004664	
NEAR_BAIT_BASES	1140944937	1055499631	1457363723	1593620365	1549648933	1128162517	1025319620	938744620	
OFF_BAIT_BASES	334495460	309426314	430910838	515079432	492134744	336569900	287226346	279947936	
ON_TARGET_BASES	2440842236	2244920309	3201046680	4105730234	3498398004	2511802216	2260953633	2144004664	
PCT_SELECTED_BASES	0.914589	0.914283	0.91533	0.917116	0.91117	0.915361	0.919623	0.916749	
PCT_OFF_BAIT	0.085411	0.085717	0.08467	0.082884	0.08883	0.084639	0.080377	0.083251	
ON_BAIT_VS_SELECTED	0.681459	0.680192	0.687154	0.720386	0.69302	0.690062	0.687999	0.695485	
MEAN_BAIT_COVERAGE	47.160454	43.374971	61.848658	79.328398	67.593897	48.5315	43.684757	41.425141	
MEAN_TARGET_COVERAGE	49.941324	45.938688	64.820174	82.918804	70.849348	51.2838	46.069406	43.667083	**56.9360**
PCT_USABLE_BASES_ON_BAIT	0.441695	0.436691	0.409099	0.366793	0.409759	0.440871	0.453786	0.430487	
PCT_USABLE_BASES_ON_TARGET	0.441695	0.436691	0.409099	0.366793	0.409759	0.440871	0.453786	0.430487	
FOLD_ENRICHMENT	37.278805	37.197052	37.620836	39.517131	37.769523	37.781306	37.843754	38.135922	
ZERO_CVG_TARGETS_PCT	0.068777	0.069162	0.057681	0.054544	0.05756	0.066934	0.064603	0.064252	
FOLD_80_BASE_PENALTY	4.161777	4.176244	4.321345	3.948514	3.728913	4.662164	4.188128	3.969735	
PCT_TARGET_BASES_2X	0.884022	0.884797	0.900662	0.909093	0.904828	0.886326	0.889255	0.890892	
PCT_TARGET_BASES_10X	0.77914	0.773672	0.811821	0.837209	0.829908	0.775414	0.779082	0.778127	**0.7955**

PCT_TARGET_BASES_20X	0.670376	0.657514	0.72642	0.773032	0.756544	0.66737	0.660251	0.65157
PCT_TARGET_BASES_30X	0.561447	0.54183	0.642268	0.710438	0.677215	0.564964	0.541801	0.525149
HS_LIBRARY_SIZE	201999973	176067418	185131944	146547112	213771006	192693824	191301053	137688765
HS_PENALTY_10X	7.565977	7.768194	7.939897	6.992338	6.554674	8.547061	7.558408	7.453889
HS_PENALTY_20X	8.880657	9.443108	9.61526	8.697407	7.453917	10.399736	8.987081	9.686605
HS_PENALTY_30X	11.159436	12.912903	13.008943	12.751347	8.793049	14.300646	11.589813	17.549268

Table S2: **Filtered somatic mutation candidates identified by whole exome sequencing in 4 IDH-mutant LGGs.**

Tumor	Chr	Start Position	End Position	Ref Allele	Tumor Allele	Gene	Mutation Type
G4	chr11	73105299	73105299	A	G	RELT	nonsynonymous SNV
G4	chr13	29933470	29933470	G	A	MTUS2	nonsynonymous SNV
G4	chr1	32264124	32264124	C	A	SPOCD1	nonsynonymous SNV
G4	chr8	1574980	1574980	C	A	DLGAP2	nonsynonymous SNV
G4	chr17	26907057	26907057	C	T	SPAG5	nonsynonymous SNV
G4	chr17	7573988	7573988	C	T	TP53	nonsynonymous SNV
G4	chr16	3293978	3293978	G	A	MEFV	nonsynonymous SNV
G4	chr13	110817254	110817254	C	G	COL4A1	nonsynonymous SNV
G4	chr3	124560357	124560357	C	T	ITGB5	nonsynonymous SNV
G4	chr9	67930409	67930409	A	G	ANKRD20A1	nonsynonymous SNV
G4	chr19	15297966	15297966	C	G	NOTCH3	nonsynonymous SNV
G4	chr12	121476582	121476582	C	G	OASL	nonsynonymous SNV
G4	chr15	40558665	40558665	G	A	PAK6	nonsynonymous SNV
G4	chrX	134156272	134156272	G	T	FAM127C	nonsynonymous SNV
G4	chr1	144828707	144828707	G	T	NBPF9	nonsynonymous SNV
G4	chr9	35698501	35698501	G	A	TLN1	nonsynonymous SNV
G4	chr15	76566799	76566799	A	G	ETFA	nonsynonymous SNV
G4	chrX	153633801	153633801	C	G	DNASE1L1	nonsynonymous SNV
G4	chr9	139413166	139413166	C	T	NOTCH1	nonsynonymous SNV
G4	chr2	219925077	219925077	C	G	IHH	nonsynonymous SNV
G4	chr15	56132689	56132689	T	C	NEDD4	nonsynonymous SNV
G4	chr19	20045209	20045209	A	G	ZNF93	nonsynonymous SNV

G4	chrX	47755285	47755285	C	A	ZNF81	nonsynonymous SNV
G4	chr2	209113112	209113112	C	T	IDH1	nonsynonymous SNV
G4	chr17	38183116	38183116	T	C	MED24	nonsynonymous SNV
G4	chr11	6239278	6239278	C	A	FAM160A2	nonsynonymous SNV
G4	chr18	72999306	72999306	C	T	TSHZ1	nonsynonymous SNV
G4	chr2	206892854	206892854	C	A	INO80D	nonsynonymous SNV
G4	chr1	19181060	19181060	C	G	TAS1R2	nonsynonymous SNV
G4	chr3	195514585	195514585	G	A	MUC4	nonsynonymous SNV
G4	chr1	175048754	175048754	G	T	TNN	nonsynonymous SNV
G4	chr2	233244536	233244536	G	A	ALPP	nonsynonymous SNV
G4	chr1	172431340	172431340	T	C	C1orf105	nonsynonymous SNV
G4	chrX	114426538	114426538	C	T	RBMXL3	nonsynonymous SNV
G4	chr7	72892752	72892752	T	C	BAZ1B	nonsynonymous SNV
G4	chr10	100167674	100167674	C	A	PYROXD2	nonsynonymous SNV
G4	chr17	72860286	72860286	G	T	FDXR	nonsynonymous SNV
G4	chr9	131300364	131300364	C	A	GLE1	nonsynonymous SNV
G4	chr19	1036169	1036169	T	A	CNN2	nonsynonymous SNV
G4	chr5	176795201	176795201	G	T	RGS14	nonsynonymous SNV
G4	chr11	17531367	17531367	G	C	USH1C	nonsynonymous SNV
G4	chr5	141233987	141233987	G	A	PCDH1	nonsynonymous SNV
G4	chr22	47068794	47068794	C	G	GRAMD4	nonsynonymous SNV
G4	chr18	44140203	44140203	C	A	LOXHD1	nonsynonymous SNV
G4	chr22	36692963	36692963	C	A	MYH9	nonsynonymous SNV
G4	chr9	138387401	138387401	G	A	C9orf116	nonsynonymous SNV
G4	chr22	38461028	38461028	C	A	PICK1	nonsynonymous SNV
G4	chr12	133779878	133779878	G	T	ZNF268	nonsynonymous SNV
G4	chr5	140250385	140250385	C	T	PCDHA11	nonsynonymous SNV
G4	chr15	43925101	43925101	A	C	CATSPER2	nonsynonymous SNV
G4	chrX	108780221	108780221	G	A	NXT2	nonsynonymous SNV
G4	chr17	9511516	9511516	C	A	WDR16	nonsynonymous SNV
G4	chr16	823026	823026	G	A	MSLNL	nonsynonymous SNV
G4	chr2	219920500	219920500	G	A	IHH	nonsynonymous SNV
G4	chr5	121187845	121187845	G	T	FTMT	nonsynonymous SNV
G4	chr11	118484597	118484597	C	G	PHLDB1	nonsynonymous SNV
G4	chr7	2978376	2978376	C	A	CARD11	nonsynonymous SNV
G4	chr19	3614534	3614534	A	T	C19orf29	nonsynonymous SNV

G4	chr8	11301797	11301797	G	A	FAM167A	nonsynonymous SNV
G4	chr3	75787864	75787864	T	A	ZNF717	nonsynonymous SNV
G4	chr22	43687069	43687069	C	T	SCUBE1	nonsynonymous SNV
G4	chr12	122669189	122669189	C	T	LRRC43	nonsynonymous SNV
G4	chr14	52485857	52485857	C	T	NID2	nonsynonymous SNV
G4	chr1	32713192	32713192	G	C	FAM167B	nonsynonymous SNV
G4	chr3	132440955	132440955	G	T	NPHP3	nonsynonymous SNV
G4	chr3	10417322	10417322	A	G	ATP2B2	nonsynonymous SNV
G4	chr17	4455494	4455494	C	G	MYBBP1A	nonsynonymous SNV
G4	chr1	15909879	15909879	C	T	AGMAT	nonsynonymous SNV
G4	chr9	97869409	97869409	A	G	FANCC	nonsynonymous SNV
G4	chr13	52509103	52509103	G	A	ATP7B	nonsynonymous SNV
G4	chr9	127245116	127245116	T	C	NR5A1	nonsynonymous SNV
G4	chr10	37482163	37482163	T	A	ANKRD30A	nonsynonymous SNV
G4	chr19	17688845	17688845	C	A	GLT25D1	nonsynonymous SNV
G4	chr19	39888332	39888332	C	T	MED29	nonsynonymous SNV
G4	chr17	39411903	39411903	G	C	KRTAP9-9	nonsynonymous SNV
G4	chr11	62458330	62458330	C	A	BSCL2	nonsynonymous SNV
G4	chr10	13701380	13701380	C	T	FRMD4A	nonsynonymous SNV
G4	chr15	42179639	42179639	C	G	SPTBN5	nonsynonymous SNV
G4	chr19	41029461	41029461	G	T	SPTBN4	nonsynonymous SNV
G4	chr1	52499135	52499135	G	A	KTI12	nonsynonymous SNV
G4	chr10	48429459	48429459	G	A	GDF10	nonsynonymous SNV
G4	chr3	75786288	75786288	A	G	ZNF717	nonsynonymous SNV
G5	chr14	75248079	75248079	C	A	YLPM1	nonsynonymous SNV
G5	chr6	32551949	32551949	C	G	HLA-DRB1	nonsynonymous SNV
G5	chr15	89400002	89400002	T	G	ACAN	nonsynonymous SNV
G5	chr20	62120457	62120457	C	T	EEF1A2	nonsynonymous SNV
G5	chr1	228401878	228401878	A	G	OBSCN	nonsynonymous SNV
G5	chr19	22156758	22156758	T	C	ZNF208	nonsynonymous SNV
G5	chr17	73909996	73909996	G	T	FBF1	nonsynonymous SNV
G5	chr10	94645	94645	C	G	TUBB8	nonsynonymous SNV
G5	chr19	36002431	36002431	C	T	DMKN	nonsynonymous SNV
G5	chr5	1501639	1501639	G	C	LPCAT1	nonsynonymous SNV
G5	chr22	51133361	51133361	C	G	SHANK3	nonsynonymous SNV
G5	chr9	131197984	131197984	C	G	CERCAM	nonsynonymous SNV

G5	chr9	33264299	33264299	T	C	BAG1	nonsynonymous SNV
G5	chr1	148594563	148594563	G	T	NBPF15	nonsynonymous SNV
G5	chr1	110034112	110034112	T	C	ATXN7L2	nonsynonymous SNV
G5	chr1	248525639	248525639	A	C	OR2T4	nonsynonymous SNV
G5	chr1	6523132	6523132	T	C	TNFRSF25	nonsynonymous SNV
G5	chr2	209113113	209113113	G	C	IDH1	nonsynonymous SNV
G5	chr22	38474697	38474697	C	T	SLC16A8	nonsynonymous SNV
G5	chr7	100644624	100644624	A	T	MUC12	nonsynonymous SNV
G5	chr17	38347859	38347859	T	C	RAPGEFL1	nonsynonymous SNV
G5	chr17	7577121	7577121	G	A	TP53	nonsynonymous SNV
G5	chr14	24795075	24795075	C	A	ADCY4	nonsynonymous SNV
G5	chr20	4228530	4228530	G	A	ADRA1D	nonsynonymous SNV
G5	chr22	19967727	19967727	G	A	ARVCF	nonsynonymous SNV
G5	chrY	15019451	15019451	G	C	DDX3Y	nonsynonymous SNV
G5	chrX	152815514	152815514	G	T	ATP2B3	nonsynonymous SNV
G5	chr17	4844266	4844266	C	A	RNF167	nonsynonymous SNV
G5	chr15	65625687	65625687	A	G	IGDCC3	nonsynonymous SNV
G5	chr19	11289229	11289229	C	A	KANK2	nonsynonymous SNV
G5	chr9	117046041	117046041	A	C	COL27A1	nonsynonymous SNV
G5	chr19	1058623	1058623	G	A	ABCA7	nonsynonymous SNV
G5	chr15	26866488	26866488	G	A	GABRB3	nonsynonymous SNV
G5	chr22	37628855	37628855	A	G	RAC2	nonsynonymous SNV
G5	chr3	113673175	113673175	T	C	ZDHHC23	nonsynonymous SNV
G5	chr16	22545979	22545979	C	G	LOC100132247	nonsynonymous SNV
G5	chr7	150831635	150831635	C	A	AGAP3	nonsynonymous SNV
G5	chr4	15067992	15067992	C	G	CPEB2	nonsynonymous SNV
G5	chr15	38786776	38786776	G	A	RASGRP1	nonsynonymous SNV
G5	chr2	219825176	219825176	T	C	CDK5R2	nonsynonymous SNV
G5	chrX	65420541	65420541	C	G	HEPH	nonsynonymous SNV
G5	chr22	43026916	43026916	T	C	CYB5R3	nonsynonymous SNV
G5	chr21	34948686	34948686	G	A	SON	nonsynonymous SNV
G5	chr17	7467018	7467018	G	A	SENP3	nonsynonymous SNV
G5	chr15	89400003	89400003	C	G	ACAN	nonsynonymous SNV
G5	chr1	44402402	44402402	C	T	ARTN	nonsynonymous SNV
G5	chr1	22172706	22172706	C	A	HSPG2	nonsynonymous SNV
G5	chr11	93416774	93416774	T	C	KIAA1731	nonsynonymous SNV

G5	chr8	48842561	48842561	G	A	PRKDC	nonsynonymous SNV
G5	chr1	148024831	148024831	C	G	NBPF14	nonsynonymous SNV
G5	chr9	34724869	34724869	C	T	FAM205A	nonsynonymous SNV
G5	chr20	47249148	47249148	T	C	PREX1	nonsynonymous SNV
G5	chr21	33732214	33732214	A	G	URB1	nonsynonymous SNV
G5	chr1	1849862	1849862	A	G	TMEM52	nonsynonymous SNV
G5	chr10	50022092	50022092	C	T	WDFY4	nonsynonymous SNV
G5	chr12	50190435	50190435	A	G	NCKAP5L	nonsynonymous SNV
G5	chr3	195514214	195514214	C	T	MUC4	nonsynonymous SNV
G5	chr19	40368418	40368418	G	T	FCGBP	nonsynonymous SNV
G5	chr19	7056758	7056758	G	T	MBD3L3	nonsynonymous SNV
G5	chr15	74325753	74325753	C	T	PML	nonsynonymous SNV
G5	chr14	67647602	67647602	T	C	GPHN	nonsynonymous SNV
G5	chr19	6010206	6010206	A	G	RFX2	nonsynonymous SNV
G5	chr2	130872791	130872791	A	G	POTEF	nonsynonymous SNV
G5	chr5	178460520	178460520	G	T	ZNF879	nonsynonymous SNV
G5	chr1	220101212	220101212	C	T	SLC30A10	nonsynonymous SNV
G5	chr9	132897358	132897358	C	A	GPR107	nonsynonymous SNV
G5	chr19	6427150	6427150	G	T	SLC25A41	nonsynonymous SNV
G5	chr1	41976933	41976933	T	G	HIVEP3	nonsynonymous SNV
G5	chr12	95445686	95445686	C	T	NR2C1	nonsynonymous SNV
G5	chr1	225533774	225533774	C	A	DNAH14	nonsynonymous SNV
G5	chr16	58030656	58030656	C	T	ZNF319	nonsynonymous SNV
G5	chr19	41381632	41381632	G	C	CYP2A7	nonsynonymous SNV
G5	chr9	135983613	135983613	T	C	RALGDS	nonsynonymous SNV
G5	chr3	183960728	183960728	A	G	ALG3	nonsynonymous SNV
G5	chrX	49083113	49083113	A	G	CACNA1F	nonsynonymous SNV
G5	chr9	127230066	127230066	G	A	GPR144	nonsynonymous SNV
G5	chr19	1117395	1117395	A	G	SBNO2	nonsynonymous SNV
G5	chr22	18314730	18314730	T	C	MICAL3	nonsynonymous SNV
G5	chr8	12863782	12863782	T	A	KIAA1456	nonsynonymous SNV
G5	chr1	154962897	154962897	G	A	FLAD1	nonsynonymous SNV
G5	chr17	60767541	60767541	G	A	MRC2	nonsynonymous SNV
G5	chrX	48978788	48978788	G	T	GPKOW	nonsynonymous SNV
G5	chrX	92927123	92927123	C	A	NAP1L3	nonsynonymous SNV
G5	chrX	89177635	89177635	C	A	TGIF2LX	nonsynonymous SNV

G5	chr16	4752181	4752181	C	T	ANKS3	nonsynonymous SNV
G5	chr1	159902334	159902334	C	T	IGSF9	nonsynonymous SNV
G5	chr7	27194580	27194580	T	C	HOXA7	nonsynonymous SNV
G5	chr1	33235649	33235649	C	G	KIAA1522	nonsynonymous SNV
G5	chr11	44129655	44129655	T	G	EXT2	nonsynonymous SNV
G5	chr17	48680553	48680553	C	G	CACNA1G	nonsynonymous SNV
G5	chr15	28437270	28437270	G	C	HERC2	nonsynonymous SNV
G5	chr9	33264300	33264300	C	T	BAG1	nonsynonymous SNV
G5	chr3	49700545	49700545	C	T	BSN	nonsynonymous SNV
G5	chr17	15620009	15620009	G	A	ZNF286A	nonsynonymous SNV
G5	chr11	119058332	119058332	G	T	PDZD3	nonsynonymous SNV
G5	chr5	6449287	6449287	C	A	UBE2QL1	nonsynonymous SNV
G5	chr5	1074784	1074784	G	A	SLC12A7	nonsynonymous SNV
G5	chrX	107979055	107979055	C	A	IRS4	nonsynonymous SNV
G5	chr4	57189669	57189669	C	T	KIAA1211	nonsynonymous SNV
G5	chr20	62120453	62120453	A	G	EEF1A2	nonsynonymous SNV
G5	chr22	50703381	50703381	T	C	MAPK11	nonsynonymous SNV
G5	chr7	100641088	100641088	C	T	MUC12	nonsynonymous SNV
G5	chr16	14970361	14970361	C	A	NOMO1	nonsynonymous SNV
G5	chr13	106142411	106142411	C	A	DAOA	nonsynonymous SNV
G5	chr17	48628425	48628425	G	A	SPATA20	nonsynonymous SNV
G5	chr6	32551948	32551948	G	C	HLA-DRB1	nonsynonymous SNV
G18	chr8	72977777	72977777	C	T	TRPA1	nonsynonymous SNV
G18	chr1	152276231	152276231	G	T	FLG	nonsynonymous SNV
G18	chr11	49196449	49196449	T	C	FOLH1	nonsynonymous SNV
G18	chr7	47879087	47879087	T	C	PKD1L1	nonsynonymous SNV
G18	chr14	23829927	23829927	C	T	EFS	nonsynonymous SNV
G18	chr19	21476393	21476393	A	T	ZNF708	nonsynonymous SNV
G18	chr17	4451541	4451541	A	T	MYBBP1A	nonsynonymous SNV
G18	chr19	58499659	58499659	C	T	ZNF606	nonsynonymous SNV
G18	chr16	48234267	48234267	C	T	ABCC11	nonsynonymous SNV
G18	chr1	25890257	25890257	C	A	LDLRAP1	nonsynonymous SNV
G18	chr9	33264299	33264299	T	C	BAG1	nonsynonymous SNV
G18	chr10	104416749	104416749	G	A	TRIM8	nonsynonymous SNV
G18	chr16	31092038	31092038	T	C	ZNF646	nonsynonymous SNV
G18	chr16	67685908	67685908	G	T	RLTPR	nonsynonymous SNV

G18	chr4	156832695	156832695	T	G	TDO2	nonsynonymous SNV
G18	chr3	127337871	127337871	A	G	MCM2	nonsynonymous SNV
G18	chr3	126730984	126730984	C	G	PLXNA1	nonsynonymous SNV
G18	chr2	209113112	209113112	C	T	IDH1	nonsynonymous SNV
G18	chr22	36678799	36678799	C	G	MYH9	nonsynonymous SNV
G18	chr19	59069703	59069703	A	C	UBE2M	nonsynonymous SNV
G18	chr2	27668237	27668237	T	C	IFT172	nonsynonymous SNV
G18	chr17	61621402	61621402	C	T	KCNH6	nonsynonymous SNV
G18	chr9	130507324	130507324	G	A	SH2D3C	nonsynonymous SNV
G18	chr1	151260733	151260733	C	T	ZNF687	nonsynonymous SNV
G18	chr3	114070191	114070191	G	A	ZBTB20	nonsynonymous SNV
G18	chr22	36897395	36897395	C	T	FOXRED2	nonsynonymous SNV
G18	chr11	61503253	61503253	G	A	DAGLA	nonsynonymous SNV
G18	chr8	119936828	119936828	A	C	TNFRSF11B	nonsynonymous SNV
G18	chr16	67579636	67579636	A	G	FAM65A	nonsynonymous SNV
G18	chr9	69423863	69423863	T	A	ANKRD20A4	nonsynonymous SNV
G18	chr1	113255054	113255054	C	T	PPM1J	nonsynonymous SNV
G18	chr9	96021681	96021681	C	G	WNK2	nonsynonymous SNV
G18	chr19	53787734	53787734	G	C	LOC646508	nonsynonymous SNV
G18	chr2	108487999	108487999	A	G	RGPD4	nonsynonymous SNV
G18	chr6	27925370	27925370	A	G	OR2B6	nonsynonymous SNV
G18	chr10	94828227	94828227	C	A	CYP26C1	nonsynonymous SNV
G18	chr4	13383173	13383173	C	T	RAB28	nonsynonymous SNV
G18	chr6	168366443	168366443	C	G	MLLT4	nonsynonymous SNV
G18	chr19	40519663	40519663	T	A	ZNF546	nonsynonymous SNV
G18	chr17	7259572	7259572	G	C	TMEM95	nonsynonymous SNV
G18	chr9	33264300	33264300	C	T	BAG1	nonsynonymous SNV
G18	chr16	85114956	85114956	A	G	KIAA0513	nonsynonymous SNV
G18	chr19	3746495	3746495	C	A	TJP3	nonsynonymous SNV
G18	chr1	228511047	228511047	A	T	OBSCN	nonsynonymous SNV
G18	chr9	96422757	96422757	C	A	PHF2	nonsynonymous SNV
G18	chrX	100076521	100076521	A	G	CSTF2	nonsynonymous SNV
G18	chr16	10971191	10971191	C	A	CIITA	nonsynonymous SNV
G18	chrX	152858156	152858156	C	A	FAM58A	nonsynonymous SNV
G18	chr11	64074707	64074707	G	A	ESRRA	nonsynonymous SNV
G18	chr2	86333393	86333393	G	T	PTCD3	nonsynonymous SNV

G18	chr20	60791441	60791441	A	G	HRH3	nonsynonymous SNV
G18	chr22	51121841	51121841	T	C	SHANK3	nonsynonymous SNV
G18	chr1	39878662	39878662	G	A	KIAA0754	nonsynonymous SNV
G18	chr11	74904518	74904518	C	T	SLCO2B1	nonsynonymous SNV
G18	chr13	99093076	99093076	A	G	FARP1	nonsynonymous SNV
G18	chr17	7637506	7637506	C	T	DNAH2	nonsynonymous SNV
G18	chr1	84880381	84880381	G	A	DNASE2B	nonsynonymous SNV
G18	chr14	105395093	105395093	C	A	PLD4	nonsynonymous SNV
G18	chr2	96781258	96781258	C	T	ADRA2B	nonsynonymous SNV
G18	chr17	61607853	61607853	C	T	KCNH6	nonsynonymous SNV
G18	chr2	130878055	130878055	A	C	POTEF	nonsynonymous SNV
G6	chr16	21415837	21415837	C	T	NPIPL3	nonsynonymous SNV
G6	chr19	21607526	21607526	A	G	ZNF493	nonsynonymous SNV
G6	chr19	51958020	51958020	C	A	SIGLEC8	nonsynonymous SNV
G6	chr16	57075470	57075470	G	A	NLRC5	nonsynonymous SNV
G6	chr11	3111847	3111847	G	T	OSBPL5	nonsynonymous SNV
G6	chr7	44805769	44805769	G	A	ZMIZ2	nonsynonymous SNV
G6	chr19	45404543	45404543	C	T	TOMM40	nonsynonymous SNV
G6	chr19	15569347	15569347	C	A	RASAL3	nonsynonymous SNV
G6	chr10	129676535	129676535	C	T	CLRN3	nonsynonymous SNV
G6	chr16	67866389	67866389	G	A	CENPT	nonsynonymous SNV
G6	chr14	24601017	24601017	G	A	FITM1	nonsynonymous SNV
G6	chr19	55147967	55147967	C	T	LILRB1	nonsynonymous SNV
G6	chr2	220250367	220250367	G	A	DNPEP	nonsynonymous SNV
G6	chr1	145328389	145328389	C	G	NBPF10	nonsynonymous SNV
G6	chr19	40704322	40704322	C	A	MAP3K10	nonsynonymous SNV
G6	chr11	47609666	47609666	C	T	FAM180B	nonsynonymous SNV
G6	chr12	6960102	6960102	C	A	CDCA3	nonsynonymous SNV
G6	chr6	32729439	32729439	T	C	HLA-DQB2	nonsynonymous SNV
G6	chr17	34325937	34325937	G	A	CCL15	nonsynonymous SNV
G6	chr1	207793287	207793287	G	T	CR1	nonsynonymous SNV
G6	chrX	152226781	152226781	C	A	PNMA3	nonsynonymous SNV
G6	chr1	152187981	152187981	G	A	HRNR	nonsynonymous SNV
G6	chr11	60702808	60702808	G	A	TMEM132A	nonsynonymous SNV
G6	chr3	17446233	17446233	C	A	TBC1D5	nonsynonymous SNV
G6	chr7	158534524	158534524	G	A	ESYT2	nonsynonymous SNV

G6	chr10	71851577	71851577	C	G	H2AFY2	nonsynonymous SNV
G6	chr17	7577121	7577121	G	A	TP53	nonsynonymous SNV
G6	chr2	209113112	209113112	C	T	IDH1	nonsynonymous SNV
G6	chr19	2853583	2853583	T	C	ZNF555	nonsynonymous SNV
G6	chr21	44838355	44838355	G	A	SIK1	nonsynonymous SNV
G6	chr3	48462284	48462284	G	A	PLXNB1	nonsynonymous SNV
G6	chr19	51453207	51453207	C	A	KLK5	nonsynonymous SNV
G6	chr8	145057709	145057709	A	G	PARP10	nonsynonymous SNV
G6	chr2	120231120	120231120	C	T	SCTR	nonsynonymous SNV
G6	chr20	62421617	62421617	C	A	ZBTB46	nonsynonymous SNV
G6	chr19	42879986	42879986	C	A	MEGF8	nonsynonymous SNV
G6	chr7	44805006	44805006	C	A	ZMIZ2	nonsynonymous SNV
G6	chr15	90016033	90016033	G	A	RHCG	nonsynonymous SNV
G6	chr2	228131171	228131171	G	C	COL4A3	nonsynonymous SNV
G6	chr22	50967656	50967656	C	G	TYMP	nonsynonymous SNV
G6	chr19	36332727	36332727	C	T	NPHS1	nonsynonymous SNV
G6	chr16	71482897	71482897	C	G	ZNF23	nonsynonymous SNV
G6	chr3	128890358	128890358	G	C	CNBP	nonsynonymous SNV
G6	chr3	195507733	195507733	C	A	MUC4	nonsynonymous SNV
G6	chrX	118242329	118242329	C	A	KIAA1210	nonsynonymous SNV
G6	chr19	39111041	39111041	T	C	EIF3K	nonsynonymous SNV
G6	chr17	2966250	2966250	A	G	OR1D5	nonsynonymous SNV
G6	chr19	18542851	18542851	C	A	SSBP4	nonsynonymous SNV
G6	chr4	86332	86332	A	C	ZNF595	nonsynonymous SNV
G6	chr14	105618064	105618064	C	T	JAG2	nonsynonymous SNV
G6	chr11	6566458	6566458	A	G	DNHD1	nonsynonymous SNV
G6	chr16	65038741	65038741	A	G	CDH11	nonsynonymous SNV
G6	chr21	37618511	37618511	G	T	DOPEY2	nonsynonymous SNV
G6	chr2	96949592	96949592	G	A	SNRNP200	nonsynonymous SNV
G6	chrX	152087589	152087589	A	T	ZNF185	nonsynonymous SNV
G6	chr20	44578183	44578183	C	A	ZNF335	nonsynonymous SNV
G6	chr19	30199181	30199181	C	T	C19orf12	nonsynonymous SNV
G6	chr22	38013036	38013036	C	A	GGA1	nonsynonymous SNV
G6	chr1	165177361	165177361	C	G	LMX1A	nonsynonymous SNV
G6	chr15	24923230	24923230	C	T	C15orf2	nonsynonymous SNV
G6	chr18	3214971	3214971	C	T	MYOM1	nonsynonymous SNV

G6	chr2	120438965	120438965	G	T	TMEM177	nonsynonymous SNV
G6	chr19	44097404	44097404	C	A	IRGQ	nonsynonymous SNV
G6	chr6	158923594	158923594	C	A	TULP4	nonsynonymous SNV
G6	chr19	20727481	20727481	T	G	ZNF737	nonsynonymous SNV
G6	chr17	80146158	80146158	T	C	CCDC57	nonsynonymous SNV
G6	chr10	81706408	81706408	A	G	SFTPD	nonsynonymous SNV
G6	chr11	67270108	67270108	C	A	PITPNM1	nonsynonymous SNV
G6	chr8	22414397	22414397	C	A	SORBS3	nonsynonymous SNV
G6	chr12	122359384	122359384	G	A	WDR66	nonsynonymous SNV
G6	chr12	122359392	122359392	G	A	WDR66	nonsynonymous SNV
G4	chr4	123234782	123234782	G	T	KIAA1109	splicing
G4	chr8	17928901	17928901	T	C	ASAH1	splicing
G4	chr9	35698501	35698501	G	A	TLN1	splicing
G4	chr2	24246452	24246452	G	A	MFSD2B	splicing
G4	chr1	209878137	209878137	G	C	HSD11B1	splicing
G4	chr2	238990850	238990850	G	A	SCLY	splicing
G4	chr3	146309505	146309505	A	G	PLSCR5	splicing
G4	chr15	42179639	42179639	C	G	SPTBN5	splicing
G5	chr1	6523132	6523132	T	C	TNFRSF25	splicing
G5	chr2	138033493	138033493	G	C	THSD7B	splicing
G5	chr6	37139268	37139268	G	T	PIM1	splicing
G5	chr19	11289229	11289229	C	A	KANK2	splicing
G5	chr9	117046041	117046041	A	C	COL27A1	splicing
G5	chr1	11009681	11009681	T	C	C1orf127	splicing
G5	chr1	41976933	41976933	T	G	HIVEP3	splicing
G5	chr10	90365388	90365388	A	T	LIPJ	splicing
G5	chr10	94822753	94822753	G	T	CYP26C1	splicing
G18	chr4	123234782	123234782	G	T	KIAA1109	splicing
G18	chr2	165984613	165984613	T	G	SCN3A	splicing
G18	chr3	127337871	127337871	A	G	MCM2	splicing
G6	chr15	41853737	41853737	C	T	TYRO3	splicing
G4	chr2	45235738	45235738	C	T	SIX2	stopgain SNV
G4	chr15	23006364	23006364	G	A	NIPA2	stopgain SNV
G5	chr16	68293443	68293443	G	A	PLA2G15	stopgain SNV
G5	chr11	45935984	45935984	G	A	PEX16	stopgain SNV
G5	chr1	36028217	36028217	G	A	NCDN	stopgain SNV

G5	chr6	43251678	43251678	C	A	TTBK1	stopgain SNV
G18	chr1	8386053	8386053	C	A	SLC45A1	stopgain SNV
G18	chr21	45797623	45797623	C	A	TRPM2	stopgain SNV
G18	chr17	2604444	2604444	G	A	KIAA0664	stopgain SNV
G18	chrX	76909629	76909629	G	A	ATRX	stopgain SNV
G18	chr13	32340147	32340147	C	A	RXFP2	stopgain SNV
G6	chr22	50660214	50660214	C	T	TUBGCP6	stopgain SNV
G6	chr22	17264513	17264513	G	C	XKR3	stopgain SNV
G6	chr20	870963	870963	G	A	ANGPT4	stopgain SNV
G6	chr9	39888046	39888046	G	T	FAM75A2	stopgain SNV
G6	chr4	8228775	8228775	C	T	SH3TC1	stopgain SNV
G6	chr2	192234306	192234306	C	G	MYO1B	stopgain SNV
G4	chr11	89774214	89774217	TGCA	-	TRIM49L2	splicing
G4	chr5	139940606	139940616	TGGCCTTGGTG	-	APBB3	frameshift deletion
G4	chr5	140953561	140953563	GGT	-	DIAPH1	nonframeshift deletion
G5	chr17	39274069	39274070	GG	-	KRTAP4-11	frameshift deletion
G5	chr17	43011619	43011620	AT	-	KIF18B	frameshift deletion
G5	chr19	55341421	55341422	AC	-	KIR3DL1	frameshift deletion
G5	chr3	49851379	49851406	GAGAAAAGAAAAGTGAAAGTTAAACTAT	-	UBA7	splicing
G5	chr3	75787099	75787100	CT	-	ZNF717	frameshift deletion
G5	chr5	139931628	139931628	-	GTC	SRA1	nonframeshift insertion
G5	chr7	142481376	142481383	TGGGGGTG	-	PRSS2	nonframeshift deletion
G5	chrX	76937745	76937745	T	-	ATRX	frameshift deletion
G18	chr11	3661583	3661583	-	GAT	ART5	nonframeshift insertion
G18	chr22	50754644	50754649	CAGGTG	-	FAM116B	nonframeshift deletion
G18	chr3	43074430	43074445	AGCTCATCAGTGGCCT	-	FAM198A	frameshift deletion
G18	chr6	32497961	32497962	TT	-	HLA-DRB5	frameshift deletion
G6	chr11	6567886	6567895	TTGAGGAGGC	-	DNHD1	frameshift deletion
G6	chr19	875243	875247	CCGAC	-	MED16	frameshift deletion
G6	chrX	76938832	76938832	A	-	ATRX	frameshift deletion

Table S3: Tabulated nucleotide transitions for point mutations seen in filtered whole exome sequencing data for 4 IDH-mutant LGGs. Reference and mutant bases are shown in the first column and row respectively.

	A	C	T	G
A		8	7	35
C	59		62	32
T	6	33		7
G	63	16	30	

Table S4: Tabulated nucleotide transitions for point mutations seen in filtered whole exome sequencing data for 4 IDH-mutant LGGs. Mutations involving either C or G transitions to either T or A predominate. Reference and mutant bases are shown in the first column and row respectively.

	T:A	C:G
T:A		83
C:G	214	

Table S5: **Numbers of unfiltered somatic variants identified by whole exome sequencing in 4 IDH-mutant LGGs.**

Tumor	SNVs	insertions/deletions	Total
G4	4408	212	4620
G5	6315	286	6601
G18	4157	176	4333
G6	4139	190	4329
Sum	19019	864	19883
Average			**4970.75**

Table S6: **Candidate somatic variants identified by whole exome sequencing in 4 IDH-mutant LGGs that were then subjected to validation by Sanger sequencing.**

Gene	Tumor	Chr Position	AA Change	Reference	Variant	Effect
TP53	G4	chr17:7573988	p.A347T	C	T	missense
TP53	G5	chr17:7577121	p.R273C	G	A	missense
TP53	G6	chr17:7577121	p.R273C	G	A	missense
IDH1	G4	chr2:209113112	p.R132H	C	T	missense
IDH1	G5	chr2:209113113	p.R132G	G	C	missense
IDH1	G18	chr2:209113112	p.R132H	C	T	missense
IDH1	G6	chr2:209113112	p.R132H	C	T	missense
MUC4	G4	chr3:195514585	p.A1289V	G	A	missense
MUC4	G5	chr3:195514214	p.D1413N	C	T	missense
MUC4	G6	chr3:195507733	p.S3573I	C	A	missense
MYH9	G4	chr22:36692963	p.E1066D	C	A	missense
MYH9	G18	chr22:36678799	p.R1933P	C	G	missense
MYBBP1A	G4	chr17:4455494	p.K277N	C	G	missense
MYBBP1A	G18	chr17:4451541	p.W541R	A	T	missense
KIAA1109	G4	chr4:123234782	NA	G	T	splicing
KIAA1109	G18	chr4:123234782	NA	G	T	splicing
BAG1	G5	chr9:33264300	p.D125N	C	T	missense
BAG1	G18	chr9:33264299	p.D125G	T	C	missense
POTEF	G5	chr2:130872791	p.I211T	A	G	missense
POTEF	G18	chr2:130878055	p.S12A	A	C	missense
SHANK3	G5	chr22:51133361	p.P397A	C	G	missense
SHANK3	G18	chr22:51121841	p.F320S	T	C	missense
NBPF14	G5	chr1:148024831	p.E56Q	C	G	missense
NBPF14	G6	chr1:148024796	p.P67P	C	T	missense
ATRX	G5	chrX:76937745	p.K1001fs	T	N/A	frameshift deletion
ATRX	G6	chrX:76938832	p.L639fs	A	N/A	frameshift deletion
ATRX	G18	chrX:76909629	p.R1426X	G	A	nonsense
OR2B6	G18	chr6:27925370	p.M118V	A	G	missense
ABCC11	G18	chr16:48234267	p.V668M	C	T	missense
GABRB3	G5	chr15:26866488	p.P145L	G	A	missense

NEDD4	G4	chr15:56132689	p.M1082V	T	C	missense
TDO2	G18	chr4:156832695	p.F222C	T	G	missense
DLGAP2	G4	chr8:1574980	p.P505Q	C	A	missense
TNFRSF11B	G18	chr8:119936828	p.L331M	A	T	missense
ITGB5	G4	chr3:124560357	p.R218H	C	T	missense
ZNF546	G18	chr19:40519663	p.S162R	T	A	missense
CR1	G6	chr1:207793287	p.V1927L	G	T	missense
KIAA1731	G5	chr11:93416774	p.L272P	T	C	missense
HERC2	G5	chr15:28437270	p.S2763C	G	C	missense
KIAA1211	G5	chr4:57189669	p.A1105V	C	T	missense
ETFA	G4	chr15:76566799	p.V257A	A	G	missense
EXT2	G5	chr11:44129655	p.N131K	T	G	missense
SRA1	G5	chr5:139931628	p.V110delinsGL	G	GTC	nonframeshift insertion

Table S7: Read number and coverage statistics for MiSeq sequencing of expansion LGG cohort and case-matched normal (N=28).

Sample	Total Reads	Mean/Base	% above 10 reads	% above 100 reads
G7 Normal	26704934	115.97	71.1	47.7
G7 Tumor	30099131	130.71	74.9	51.7
G1 Normal	27878344	121.07	72.3	50.7
G1 Tumor	16918465	73.47	72.3	29.9
G24 Normal	21375181	92.83	74.6	43.5
G24 Tumor	30969017	134.49	76.4	52.9
G3 Normal	17425444	75.67	71.4	35.9
G3 Tumor	27205600	118.14	75.2	51.2
G8 Normal	2538888	11.03	43.2	0
G8 Tumor	23174871	100.64	67.4	44.9
G26 Normal	2311587	10.04	41.7	0
G26 Tumor	29645261	128.74	70.1	50.8
G9 Normal	19597769	85.11	74	38.1
G9 Tumor	19260898	83.64	63.9	35.6
G10 Normal	16435277	71.37	70.3	32.5
G10 Tumor	21944595	95.3	63	40.6
G22 Normal	17651479	76.65	66.8	38.1
G22 Tumor	20237140	87.88	70.1	35.3
G11 Normal	15017483	65.22	62.3	32.4

G11 Tumor	29198216	126.8	75.4	50.5
G2 Normal	19109611	82.99	68.9	39.7
G2 Tumor	25657363	111.42	80.8	49.8
G27 Normal	16984467	73.76	61.9	36.4
G27 Tumor	21087345	91.58	74.4	43.5
G14 Normal	15651297	67.97	60.9	33.1
G14 Tumor	19796198	85.97	68.5	38.9
G21 Normal	15511623	67.36	70.8	31.2
G21 Tumor	24049473	104.44	67.9	42.9
G12 Normal	19856289	86.23	78.3	41.7
G12 Tumor	23838475	103.52	71.1	43.5
G31 Normal	19724762	85.66	78.6	37.7
G31 Tumor	21918440	95.18	64.4	39.8
G25 Normal	15223723	66.11	74.2	28.4
G25 Tumor	25493736	110.71	59.7	44.7
G15 Normal	19398747	84.24	74	37.2
G15 Tumor	17599539	76.43	73.2	31.8
G16 Normal	18149701	78.82	71.1	38.1
G16 Tumor	23725378	103.03	77.6	50.1
G28 Normal	14431151	62.67	60.1	28.9
G28 Tumor	17873194	77.62	81	30.7
G29 Normal	16668909	72.39	62.4	34.9
G29 Tumor	28016392	121.67	82	51.3
G30 Normal	19904354	86.44	69.6	42.2
G30 Tumor	12157396	52.8	78.5	11.6
G17 Normal	18163608	78.88	71.1	38.1
G17 Tumor	21535945	93.52	78.2	45.9
G32 Normal	17336318	75.29	74.4	34.9
G32 Tumor	25863718	112.32	77	50.2
G20 Normal	14358282	62.35	73.3	26
G20 Tumor	23479121	101.96	76.4	42.9
G19 Normal	16253201	70.58	77.2	30.3
G19 Tumor	21305825	92.52	74.5	43.1
G13 Normal	13058788	56.71	74.1	21.5
G13 Tumor	19194839	83.36	77.3	35.9
G23 Normal	15614436	67.81	70.6	30.8
G23 Tumor	15263496	66.28	75.9	24.6

Table S8: **75-gene signature (25 genes per subclass) used to develop the Nanostring classifier. NB: neuroblastic, EPL: early progenitor-like, PG: preglioblastoma, ROC: receiver operator characteristic.**

NB Genes	ROC Score	EPL Genes	ROC Score	PG Genes	ROC Score
BMP2	1.86702	PSG7	1.85817	FKBP9	1.89085
GALNT13	1.80148	KLHL14	1.84682	TAGLN2	1.8807
PCDH15	1.76824	PSG1	1.84547	VASN	1.87932
MDGA2	1.76526	CDKL2	1.83459	PDPN	1.87578
FERMT1	1.76203	KCNH5	1.82959	EMP3	1.87228
FHDC1	1.75793	POM121L10P	1.82682	ARSD	1.85618
PSITPTE22	1.74946	BPIL2	1.82209	PION	1.85452
MYC	1.74452	RNF17	1.81967	TRIP6	1.848
GSTA4	1.74179	HTR4	1.81805	RBP1	1.84417
MTPAP	1.73828	TEX13A	1.8171	NCRNA00092	1.8396
H2AFY2	1.73737	UCA1	1.81527	NR2E1	1.83522
SNHG1	1.73661	PSG4	1.81014	SLC2A10	1.83456
USP3	1.73578	WNT10B	1.80637	RAB34	1.81093
TMEM100	1.72978	KLK2	1.79624	VAV3	1.80871
HNRNPH3	1.72471	PRSS1	1.79582	MOSC2	1.80537
RCOR2	1.71932	KLK7	1.7957	SLC43A3	1.80372
CHST9	1.71447	NOX1	1.79184	PLAT	1.79212
CALCRL	1.71421	HRG	1.79063	CCDC46	1.79065
IRX2	1.71299	SPATA16	1.79021	TMEM220	1.78678
HMX1	1.70177	LMX1B	1.79002	CLEC5A	1.78586
KLRC3	1.6991	DQX1	1.78908	MT1M	1.78448
DSCAML1	1.6876	PRB3	1.78631	F11R	1.7844
KIAA0114	1.68251	BEND4	1.7811	PGCP	1.78087
ZDHHC22	1.68147	BCL2L14	1.78049	AEBP1	1.77725
KLRC4	1.67804	SLC23A1	1.77772	MAOB	1.77189
